# Transport efficiency of AtGTR1 dependents on the hydrophobicity of transported glucosinolates

**DOI:** 10.1038/s41598-022-09115-x

**Published:** 2022-03-24

**Authors:** Yi-Chia Chung, Hao-Yu Cheng, Wei-Tung Wang, Yen-Jui Chang, Shih-Ming Lin

**Affiliations:** 1 Department of Biotechnology and Bioindustry Sciences, National Cheng Kung University, Tainan, Taiwan; 2 Institute of Tropical Plant Sciences and Microbiology, National Cheng Kung University, Tainan, Taiwan

**Keywords:** Membrane proteins, Plant hormones

## Abstract

Glucosinolates (GLSs) are a group of secondary metabolites that are involved in the defense of herbivores. In *Arabidopsis thaliana*, Glucosinolate Transporter 1 (AtGTR1) transports GLSs with high affinity via a proton gradient-driven process. In addition to transporting GLSs, AtGTR1 also transports phytohormones, jasmonic acid-isoleucine (JA-Ile), and gibberellin (GA). However, little is known about the mechanisms underlying the broad substrate specificity of AtGTR1. Here, we characterized the substrate preference of AtGTR1 by using a yeast uptake assay, and the results revealed that GLS transport rates are negatively correlated with the hydrophobicity of substrates. Interestingly, the AtGTR1 showed a higher substrate affinity for GLSs with higher hydrophobicity, suggesting a hydrophobic substrate binding pocket. In addition, competition assays revealed that JA, salicylic acid (SA), and indole-3-acetic acid (IAA) competed with GLS for transport in yeast, suggesting a potential interaction of AtGTR1 with these phytohormones. To further characterize the functional properties of AtGTR1, mutagenesis experiments confirmed that the conserved EXXEK motif and Arg166 are essential for the GLS transport function. In addition, the purified AtGTR1 adopts a homodimeric conformation, which is possibly regulated by phosphorylation on Thr105. The phosphomimetic mutation, T105D, reduced its protein expression and completely abrogated its GLS transport function, indicating the essential role of phosphorylation on AtGTR1. In summary, this study investigated various factors associated with the GLS transport and increased our knowledge on the substrate preferences of AtGTR1. These findings contribute to understanding how the distribution of defense GLSs is regulated in plants and could be used to improve crop quality in agriculture.

## Introduction

Nitrate transporter 1/peptide transporter family (NPF) proteins are proton-coupled symporters that share sequence homologies with proton-dependent oligopeptide transporter (POTs) in animals and bacteria^1–3^. Unlike POTs, which primarily transport di- and tri-peptides, NPF proteins were initially identified as being involved in the regulation of nitrate uptake^1,3^. In recent years, several studies have shown that NPF proteins are also capable of transporting a variety of different substrates, such as auxin (IAA), abscisic acid (ABA), jasmonic acid (JA), gibberellins (GA), monoterpene, and glucosinolates (GLSs)^1,4–8^. These phytochemicals play essential roles in the regulation of plant growth and development, suggesting the importance of NPF proteins in plant physiology. In *Arabidopsis thaliana*, 53 members of NPF transporters are identified and divided into eight distinct subclades^9^. Among them, AtGTR1 (NPF2.10), a member of NPF2, is known to transport a variety of substrates, including GLSs, nitrate, jasmonic acid-isoleucine (JA-Ile), and GA^4,8,10,11^. It is an interesting topic to investigate how AtGTR1 exhibited a broad range of substrate specificity.

AtGTR1 is primarily expressed in the plasma membrane in the mature leaf and is also involved in regulating GLSs levels in seed^8,12^. In addition, AtGTR1 was also detected in companion cells and was found to import GLSs from the apoplast into the phloem for long-distance transport^12^. These GLSs are a group of secondary metabolites which function in herbivore defense in *Brassicaceous* plants^13,14^. GLSs can be classified into three types based on their precursors: aliphatic GLSs, derived from alanine, leucine, isoleucine, methionine or valine; benzenic GLSs, derived from phenylalanine or tyrosine; and indolic GLSs, derived from tryptophan^15,16^. AtGTR1-knockout plants showed an unbalanced distribution of all three types of GLSs, resulting in several developmental defects^8,10,17^. In vitro studies have revealed that AtGTR1 transports both aliphatic and indolic GLSs when expressed in *Xenopus* oocytes^2^. In addition, recent studies using cotton cells expressing AtGTR1 have also shown that AtGTR1 transports the benzenic sinalbin as well as the aliphatic sinigrin^18^. The molecular structures, sizes, and polarities of the three types of GLS are diverse, and the features between GLSs, JA-Ile, and GA are even more different. The critical molecular signatures of these substrates recognized by AtGTR1 are still not clear, and the mechanism underlying the broad selectivity remains to be elucidated.

The structural study of NPF6.3 (NRT1.1), a dual-affinity nitrate transporter with 28.7% identity to AtGTR1, has provided important information about the nitrate transport^19,20^. The crystal structure showed that NPF6.3 forms a homodimer, and each monomer is bound with a nitrate ion in the central cavity^19,20^. The dimeric NPF6.3 is known to be dissociated into monomers by phosphorylation of Thr101, and the dissociated NPF6.3 monomer has a high binding affinity for nitrate ions^19–21^. This phosphorylation site is also conserved as Thr105 in AtGTR1, and phosphorylation on Thr105 was found to disrupt its protein dimerization and alter its membrane localization in *Xenopus oocytes*^22^. However, it is not clear whether the dissociation of AtGTR1 affects the substrate affinity or other transport functions. In addition, another functional motif located on transmembrane helix 1 (TMH 1), EXXEK/R, is also conserved in AtGTR1 (Fig. S1). Jørgensen et al. have studied each residue of this motif in AtGTR2 and confirmed its functional role in proton translocation and active transport^23^. Although these conserved properties assist in understanding the regulatory and transporting mechanisms of AtGTR1, current studies have mainly revealed the binding sites of nitrate ions or dipeptides and therefore provide little information about the selective mechanisms for transporting GLSs and phytohormones.

To understand how plant cells specifically regulate the transport of these diverse GLSs, we aimed to identify the key factors affecting substrate selectivity of AtGTR1. Here, we expressed AtGTR1 in *Saccharomyces cerevisiae* and established a yeast uptake assay to measure the GLSs transport activities and study the substrate specificity of AtGTR1. Interestingly, we found that AtGTR1 transports all three types of GLSs and that the GLS transport rates are negatively correlated with substrate hydrophobicity. In addition, the GLSs transport activity was significantly inhibited by the addition of jasmonic acid (JA), indole-3-acetic acid (IAA), and salicylic acid (SA). Overall, this work contributes to increasing our understanding of the substrate preferences of AtGTR1 and provides a new expression system for the characterization of GLS transporters.

## Results

### AtGTR1 was localized in the plasma membrane with GLS transport activity in *S. cerevisiae*

**Figure 1:**
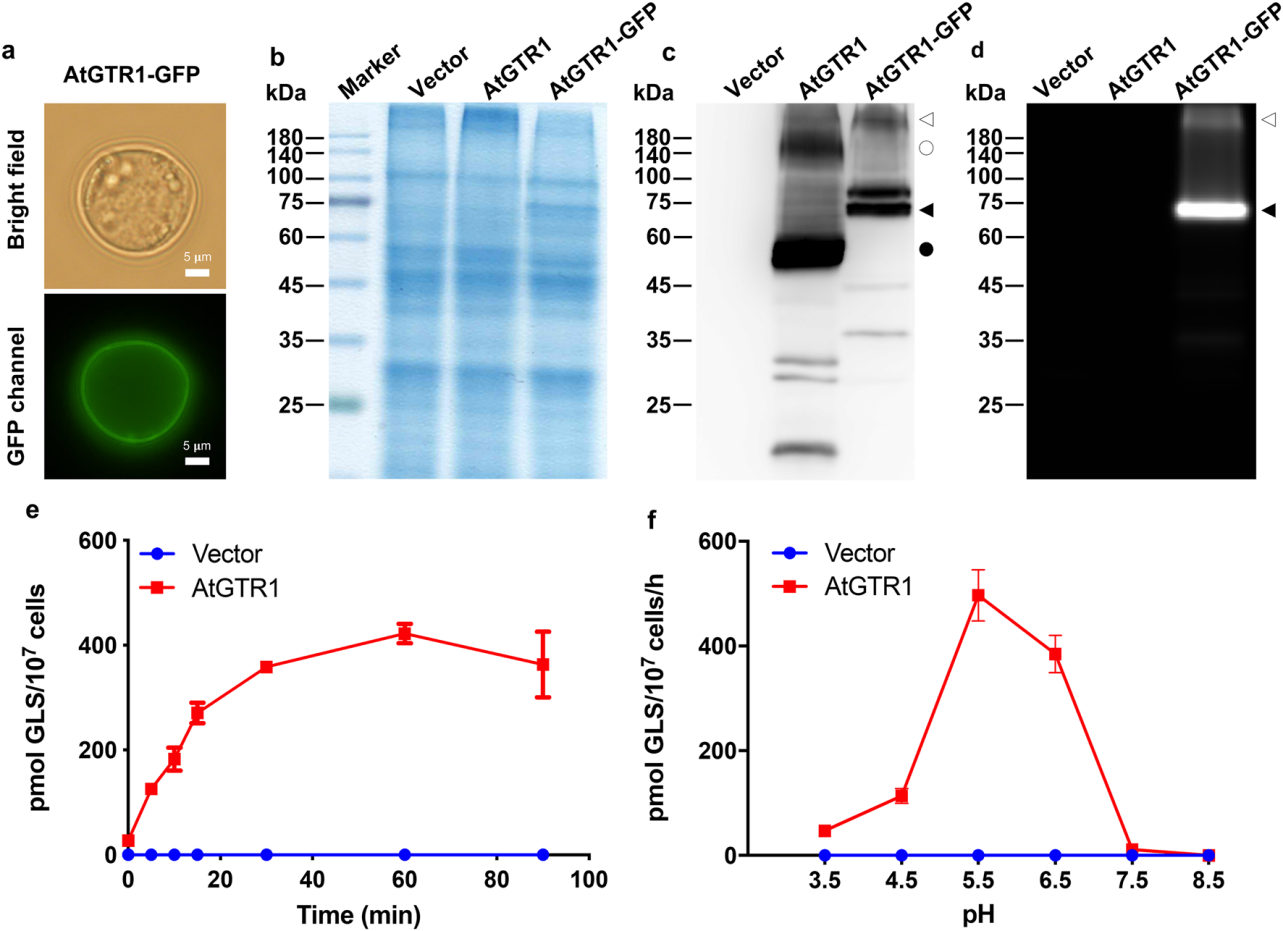
Yeast-expressed AtGTR1 localized to the cell membrane and showed the GLS transport activities. (**a**) Microscopic observation of AtGTR1-GFP-expressing yeast cells. The scale bar represents 5 μm. (**b–d**) The yeast membrane fractions isolated from AtGTR1-GFP-expressing and AtGTR1-expressing yeasts were analyzed by (**b**) SDS-PAGE, (**c**) western blotting, and (**d**) in-gel fluorescence. The primary antibody is an anti-His-tag antibody. The filled triangle and filled circle indicate the predicted positions of the AtGTR1-GFP and AtGTR1 bands, respectively. The open triangle and open circle indicate the predicted positions of dimeric AtGTR1-GFP and AtGTR1 bands, respectively. (**e**) Time-dependent GLS accumulation via AtGTR1. Yeast cells expressing AtGTR1 were incubated with 100 μM GTR for different lengths of time, and then GLS contents of the yeasts were quantified by RP-HPLC. (**f**) pH profile of the GLS transport of AtGTR1. Yeast cells were incubated in the assay medium containing 100 μM GTR with various pH for 1 h and then lysed to quantify the GLS contents by RP-HPLC. All data points with error bars represented the mean ± SD of three independent repeats.

To study the transport properties of AtGTR1, we first aimed to establish a heterologous expression system in the yeast *S. cerevisiae*. AtGTR1 fused with a c-terminal GFP was constructed to monitor the protein expression and the targeting location of AtGTR1. Figure 1a shows that AtGTR1-GFP was expressed and primarily localized at the perimeter of the yeast cells with a ring-shaped fluorescence distribution, indicating that AtGTR1 might be localized in the plasma membrane. To further confirm that AtGTR1-GFP expressed in yeast cells are membrane-localized proteins, the yeast membrane fractions were isolated and analyzed by western blot analysis with anti-His-tag antibodies and in-gel fluorescence analysis detecting GFP signals (Fig. 1b–d). The results showed that AtGTR1-GFP was present in the yeast membrane fraction with an apparent molecular weight of 75 kDa, which was slightly smaller than the theoretical size of 97 kDa (Fig. 1c,d). In addition, a minor band with a molecular weight larger than 180 kDa was detected in both western blot and in-gel fluorescence images (Fig. 1c,d), suggesting that AtGTR1-GFP may form an oligomeric conformation that is not entirely denatured in SDS-PAGE gels. Similarly, when we expressed AtGTR1 without a GFP tag in *S. cerevisiae,* AtGTR1 was detected in the yeast membrane fractions via western blot as above. Two major bands with apparent molecular sizes of 55 kDa and 140 kDa were observed (Fig. 1b,c), indicating AtGTR1 is also successfully expressed in yeast membranes and might form oligomers as AtGTR1-GFP does. Incidentally, the apparent size of AtGTR1 was also smaller than the theoretical size of 68 kDa. Similar gel shifting of membrane proteins in SDS-PAGE gels, caused by SDS-protein interactions and protein conformations, has been reported in the previous studies^24^. Thus, we concluded that both AtGTR1-GFP and AtGTR1 were expressed in yeast and primarily localized to the yeast cell membrane.

After confirming AtGTR1 expression and localization, we established a yeast uptake assay to measure the GLS transport activity and investigate the substrate specificities of AtGTR1. In this assay, yeast cells expressing AtGTR1 and vector control were incubated with GLS for different times, and then intracellular GLS was enriched by solid-state extraction and desulfated for the following quantification by RP-HPLC–DAD. LC of the control yeast cells showed a clean background without detectable GLSs (Fig. S2, middle panel), indicating that *S. cerevisiae* does not have any endogenous GLSs or GLS transporters. In contrast, GLSs substantially accumulated in yeast cells expressing AtGTR1 (Fig. S2, bottom panel). Time-course experiments indicated that the GLS content in the AtGTR1-expressing cells increased exponentially and reached saturation after 30 min, whereas the GLS level in the vector control remained non-detectable at all time points (Fig. 1e).

Next, since AtGTR1 is a symporter driven by a proton gradient, the environmental pH should affect its GLS transport activity. Although previous studies have compared the difference in transport activity of AtGTR1 at pH 5 and pH 6^18^, a more detailed pH profiling analysis is still unknown. Then, we measured the GLS transport activity of AtGTR1 at six different pH values (3.5–8.5) by using the yeast uptake assay mentioned above. The results showed that the highest GLS transport rate occurred when the pH of the medium was between 5.5 and 6.5, and the transport activity dropped sharply when the environmental pH increased from 6.5 to 7.5 (Fig. 1f). In addition, the GLS transport rate also rapidly decreased when the pH fell below 4.5. To further confirm that AtGTR1 transport activity is driven by proton gradient, the uncoupler, carbonyl cyanide m-chlorophenylhydrazone (CCCP), was applied in the yeast GLS transport assay. When the assay medium contained 10 μM CCCP, the GLS transport activity of AtGTR1 significantly decreased to about 6.4% compared to the control group (Fig. S3). These results suggest that AtGTR1 expressed in *S. cerevisiae* retains its functionality and that the yeast cell uptake assay can be utilized to measure GLS transport properties.

### AtGTR1 transports multiple types of GLSs at different rates

**Figure 2:**
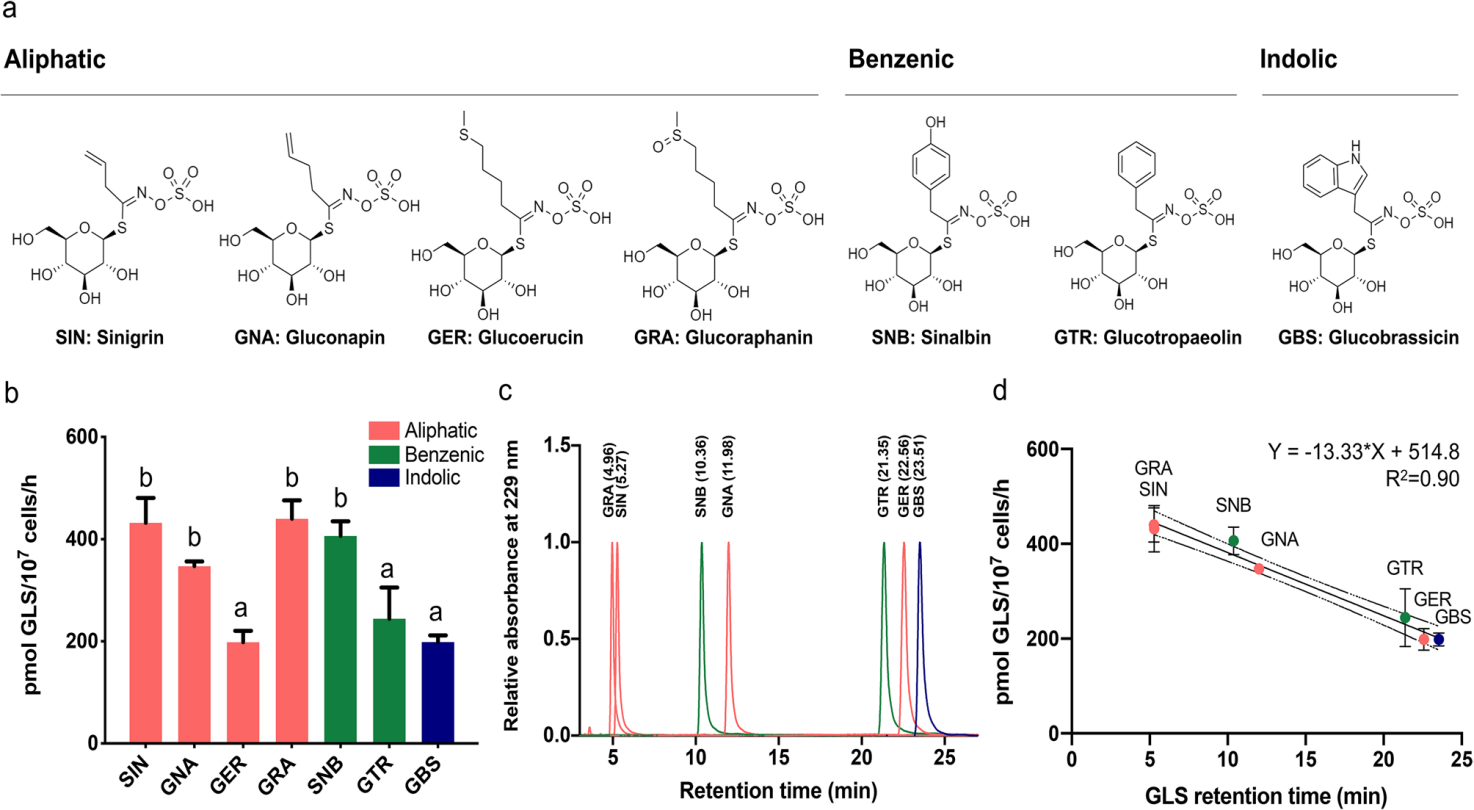
Substrate preference of AtGTR1 depends on the hydrophobicity of transported GLSs. (**a**) Molecular structures of the seven GLSs used in this study. The corresponding type is marked at the top. (**b**) Transport activity of AtGTR1 for the seven tested GLSs as measured by yeast uptake assay. The GLS transported in yeast cells were quantified by RP-HPLC. Letters above bars indicate statistically significant differences between groups (one-way ANOVA, *p* < 0.05). Bar values are the means of three independent replicates. Error bars indicate the SD. (**c**) RP-HPLC chromatogram of each desulfo-GLS standard. The compound name and retention time are marked at the top of the peaks. (**d**) The transport activity of each GLS was plotted against its retention time. The linear correlation function is shown, and the dashed line shows the confidence interval of 95%. Each data point represents mean ± SD (n = 3). The pink color represents aliphatic GLSs, the green color represents benzenic GLSs, and the blue color represents indolic GLSs.

GLSs have the same sulfonated oxime group and β-thioglucose group, but a variable aglycone side chain whose variations are based on different amino acid precursors (Fig. 2a)^13,14^. To understand the effect of various aglycone side chains on AtGTR1 transporting GLS, we selected seven GLSs, including four aliphatic GLSs, two benzenic GLSs, and one indolic GLSs, for measuring AtGTR1 transport activity. These seven GLSs are sinigrin (SIN, aliphatic), gluconapin (GNA, aliphatic), glucoerucin (GER, aliphatic), glucoraphanin (GRA, aliphatic), sinalbin (SNB, benzenic), glucotropaeolin (GTR, benzenic), and glucobrassicin (GBS, indolic), most of which are relatively abundant in *Brassica* plants (Fig. 2a)^25^. These GLSs were incubated with the yeast cells expressing AtGTR1 for one hour at a final concentration of 100 μM. The intracellular GLS contents were quantified by RP-HPLC to determine the transport rates. AtGTR1 showed varying transport rates for the uptake of these substrates but did not seem to favor any specific GLS class (Fig. 2b). Among the seven GLSs used in this study, AtGTR1 transported GRA at the highest rate, 439.9 ± 36.01 pmol/10^7^ cells/h, followed by SIN at 431.7 ± 49.09 pmol/10^7^ cells/h, SNB at 406.2 ± 28.95 pmol/10^7^ cells/h, GNA at 347.2 ± 9.20 pmol/10^7^ cells/h, GTR at 244.0 ± 61.2 pmol/10^7^ cells/h, GER at 198.0 ± 22.55 pmol/10^7^ cells/h, and GBS at 198.2 ± 13.48 pmol/10^7^ cells/h (mean ± SD, n = 3). Interestingly, we found that the transport rates of AtGTR1 for these GLSs was negatively correlated with their retention time during RP-HPLC analysis (Fig. 2c); a plot of the substrate accumulation rate and retention time showed that they shared a linear and inverse relationship, with an R squared value of 0.90 (Fig. 2d). Since the retention time of RP-HPLC correlates with the hydrophobicity of the analytes^26^, these results suggest that the transport efficiency of AtGTR1 is determined by the hydrophobicity of GLSs.

**Figure 3:**
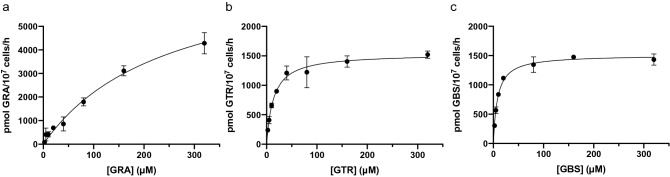
AtGTR1 showed high affinity and low efficiency for transporting GTR and GBS. The kinetic curve of AtGTR1 transporting (**a**) GRA, (**b**) GTR, and (**c**) GBS was measured by yeast uptake assay in the substrate concentration range of 2.5–320 μM. Michaelis–Menten kinetic curves were fitted using nonlinear regression, and the apparent K_m_ for GRA, GTR, and GBS are 249.2, 13.6, and 8.2 μM, respectively. Each data point represents the mean of three independent replicates (mean ± SD).

Furthermore, the kinetics of AtGTR1 transporting different GLSs was assessed, and K_m_ and V_max_ values were calculated to understand the enzymatic properties. We selected three GLSs for kinetic analysis, including aliphatic GRA, benzenic GTR, and indolic GBS. The GLS transport rate of AtGTR1 was measured using yeast uptake assay in the substrate concentration range of 2.5–320 μM and the time of substrate transport were changed to 15 min to obtain a more accurate initial rate. Figure 3 revealed that AtGTR1 showed Michaelis—Menten kinetics for transporting all GLSs in yeast. The K_m_ values of AtGTR1 are 249.2 ± 47.23 μM for GRA, 13.6 ± 1.55 μM for GTR, and 8.2 ± 0.60 μM for GBS, whereas the V_max_ values are 7677.6 ± 810.06 pmol/10^7^ cells/h for GRA, 1536.2 ± 43.73 pmol/10^7^ cells/h for GTR and 1509.8 ± 27.01 pmol/10^7^ cells/h for GBS. The higher K_m_ values suggested a lower affinity between AtGTR1 and GRA. In addition, AtGTR1 transported GRA at a much higher rate at high substrate concentrations compared to GTR and GBS. These results suggest that AtGTR1 exhibits substrate preference in terms of substrate affinity and transport efficiency. To further understand the substrate-protein interaction of AtGTR1, substrate docking models were generated using AutoDock based on the AlphaFold-predicted AtGTR1 model (Fig. S4)^27–29^. Three substrates, GRA, GTR and GBS, were selected to be docked in the central cavity of AtGTR1, respectively. The results showed that the sulfate group of GLSs interacts with the positive charged Arg166, while the aglycone side chains extend toward the hydrophobic regions in the bottom of central cavity (Fig. S4). The calculated binding energies for GRA, GTR and GBS are − 4.05, − 5.07 and − 6.18 kcal/mole, respectively. These docking simulations support the finding of kinetic experiments that AtGTR1 has a higher affinity to substrates with higher hydrophobicity.

### JA, IAA, and SA inhibited the GLS transport activity of AtGTR1 in yeast

**Figure 4:**
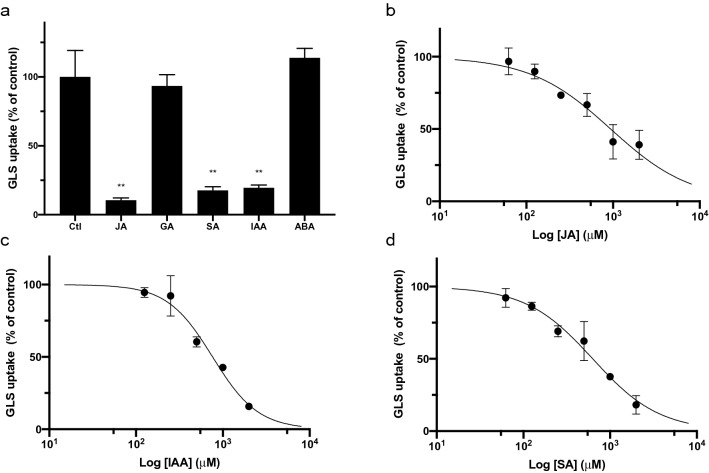
The addition of JA, IAA, and SA inhibited the transport of GLS via AtGTR1. (**a**) Yeast GLS uptake activity was evaluated in the presence of various acidic phytohormones. In each experiment, yeast cells expressing AtGTR1 were incubated with 25 μM GLS and 500 μM phytohormones for 1 h. GLS contents in the yeast cells were quantified by RP-HPLC and were normalized to the average value of the control group. *Ctl* control group, without adding phytohormones, *JA* jasmonic acid, *GA* gibberellic acid, *SA* salicylic acid, *IAA* indole-3-acetic acid, and *ABA* abscisic acid. Bar values are the means of three independent replicates. Error bars indicate SD. Significant differences were assessed by unpaired two-tailed Student t-test and are indicated by **p < 0.01. (**b–d**) Concentration–response curves for the inhibition of GLS uptake by addition of (**b**) JA, (**c**) IAA, and (**d**) SA. Various concentrations of the phytohormones were treated to inhibit GLS transport in yeast uptake assay. The GLS contents in yeast cells were quantified and normalized to the mean of the control group. Concentration–response curves were fitted using nonlinear regression. Corresponding IC_50_ values for JA, IAA, and SA are 937.8 μM, 762.1 μM and 630.0 μM, respectively. Each data point represents mean ± SD (n = 3).

In addition to transporting GLSs, AtGTR1 also transports the phytohormones, JA-Ile and GA^8,10,11^. Although these substrates share the commonality of being anions, their molecular structures are quite different from each other. It is still not clear how AtGTR1 recognizes specific phytohormones for transport, and the affinities between these phytohormones and AtGTR1 are also unknown. To answer these questions, five anionic phytohormones, including JA, GA, IAA, ABA, and SA, were selected to compete with GLSs in the yeast uptake assay. Based on the kinetic studies, GTR was chosen for these competition experiments because of its moderate K_m_ value compared to GRA and GBS. Moderate substrate affinity of GTR would be helpful to observe the competitive effects of phytohormones with weak affinity. In each experiment, 500 μM phytohormones were added against 25 μM GTR in the assay medium, and the level of reduction in the GLS transport rate was monitored. As expected, based on the reported affinity of AtGTR1 for JA, the addition of JA significantly reduced the GLS transport efficiency in the yeast uptake assay (Fig. 4a). In contrast, the addition of ABA did not affect the GLS transport significantly (Fig. 4a). Interestingly, although the previous study reports that AtGTR1 can transport GA, the addition of 500 μM GA did not reduce GLS transport into yeast cells (Fig. 4a). Unexpectedly, we found that both IAA and SA significantly inhibited GLS transport via AtGTR1 in yeast (Fig. 4a), although neither of these phytohormones has previously been reported as a substrate of AtGTR1.

To further compare the inhibitory efficiencies between JA, IAA, and SA, various concentrations of phytohormones ranging from 62.5 to 2000 µM with twofold dilution series were added against 25 µM GTR in the yeast uptake assay. The reduced GLS uptake level was quantified by RP-HPLC and normalized to the control group. The dose–response curves were fitted by nonlinear regression, and the results showed that the GLS transport activity decreased exponentially as the concentration of these phytohormones increased (Fig. 4b–d). The 50% inhibitory concentration (IC_50_) is 937.8 ± 113.85 μM for JA, 630.0 ± 47.51 μM for SA and 762.1 ± 48.14 μM for IAA (mean ± SEM, n = 3 for each concentration of competitors). Furthermore, both JA and SA showed a Hill slope near − 1 (JA: − 0.93 ± 0.120; SA: − 1.13 ± 0.100), whereas the Hill slope of IAA is − 1.66 ± 0.167, indicating IAA showed a positively cooperative inhibition. To elucidate the competitive properties of IAA, we measured the kinetic curves of AtGTR1 transporting GLS at IAA concentrations of 0, 250, 500, 1000 and 2000 μM (Fig. S5). The kinetic curves were fitted to Michaelis–Menten equation by nonlinear regression, and the V_max_ and K_m_ values are listed in Table S1. The V_max_ values decreased with increasing IAA concentration, while the K_m_ values were unaffected in presence of 250 and 500 μM IAA (Table S1). Only when the IAA concentration was higher than 1000 μM, the K_m_ value increased slightly compared to the control group (Table S1). These results suggested a mixed type, non-competitive model of IAA inhibition on the GLS transport of AtGTR1. Together, these results suggested that JA, SA, and IAA are potential substrates or analogs of AtGTR1.

### The GLS transport activity of AtGTR1 requires EXXEK motifs and Arg166 residue

**Figure 5:**
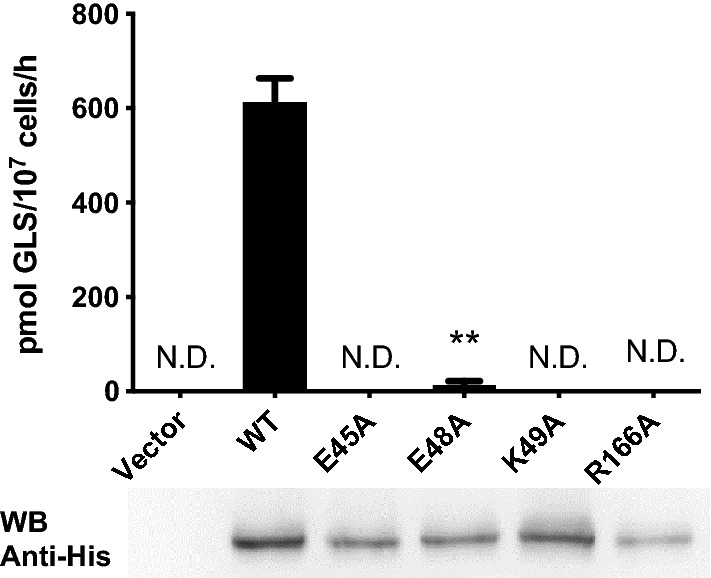
EXXEK and Arg166 are involved in the transport function of AtGTR1. The GLS transport activity of AtGTR1 mutants. The GLS transport activity was determined by yeast uptake assay, and the protein expression of these mutants was confirmed by western blot. The primary antibody is an anti-His-tag antibody. Bar values with error bar represent mean ± S.D. (n = 3); **p < 0.01; N.D. indicates nondetectable.

Previous studies have proposed the substrate-binding mechanisms for transporting nitrate ions and dipeptides in NPF and POT proteins^19,20,30^. However, the specific binding site of GLSs in AtGTR1 have rarely been experimentally confirmed. To understand how AtGTR1 recognizes GLSs, we focused on the key residues located in the central cavity of AtGTR1. Since GLSs are anionic substrates with a sulfonyl group, their negative charge should be balanced by positively charged residues during transport. Moreover, according to the modeling structure, only two positive charged residues, Lys49 and Arg166, are located in the central cavity^31^. Lys49 is part of the conserved EXXEK/R motif on TMH 1, whereas Arg166 is conserved as positively charged residues on the TMH 4 among NPF proteins (Fig. S1). To confirm that these conserved motif and residues are involved in the GLS transport of AtGTR1, we generated four alanine mutants at positions Glu43, Glu48, Lys49, and Arg166 and then observed their GLS transport activity by using yeast uptake assay. The results showed a dramatic decrease in activity for all the mutants (Fig. 5). Western blot analysis confirmed that these mutants were well expressed at the protein level, indicating that the loss of activity was not caused by an absence of expression (Fig. 5). Interestingly, of the residues in the EXXEK motif, the E48A mutant retained a very small amount of GLS transport activity, while the E45A and K49A mutants showed complete loss of function (Fig. 5). In addition, the R166A mutation also completely abrogated the GLS transport function of AtGTR1. These results suggested that the highly conserved EXXEK motif and Arg166 are essential for the GLS transport function of AtGTR1.

### AtGTR1 purified from the yeast membrane adopts a dimeric conformation

**Figure 6:**
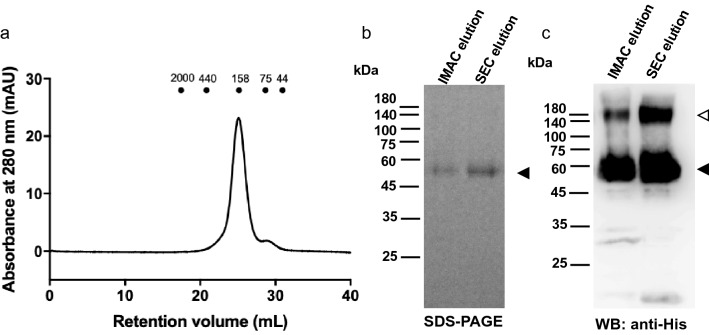
Purified AtGTR1 primarily adopts a homodimeric conformation. (**a**) SEC chromatogram of AtGTR1 showed that the major eluted peak corresponded to molecular size near 158 kDa. Points indicate the retention volume of each standard protein marker with labeled molecular sizes (kDa). (**b,c**) The AtGTR1 purified by IMAC and SEC were respectively analyzed by (**b**) SDS-PAGE and (**c**) western blot with anti-His tag antibody. The filled triangle indicates the AtGTR1 monomer, whereas the open triangle indicates the predicted dimer.

Previous studies showed that protein dimerization regulates the nitrate affinity of NPF6.3^19,21^. AtGTR1 was also known to form dimers on the plasma membrane of *Xenopus* oocytes^22^. Therefore, we want to determine the protein conformation of AtGTR1 expressed in *S. cerevisiae* to explain the GLS transport activity and selectivity observed in this study. As mentioned earlier, when analyzing the yeast membrane of AtGTR1, western blot showed a high molecular weight band, presumably a dimer of AtGTR1 (Fig. 1). To further confirm the dimerization of AtGTR1, we solubilized and purified AtGTR1 from yeast membranes by using immobilized metal affinity chromatography (IMAC), and the purified proteins were subjected to size-exclusion chromatography (SEC) to evaluate the size of the native protein complex. The major peak in the SEC chromatogram was observed at a retention volume corresponding to a molecular weight of 158 kDa, which is similar to the theoretical dimeric size of 136 kDa (Fig. 6a). In addition, a minor shoulder peak, corresponding to a molecular weight of 75 kDa, eluted next to the major peak (Fig. 6a). These purified AtGTR1 proteins were analyzed by SDS-PAGE and western blot with anti-His-tag antibody, and the results show a major band at 50 kDa and a minor band at 150 kDa in both IMAC and SEC purified products (Fig. 6b,c). Together, the results of SEC and western blot analysis showed that the majority of the AtGTR1 purified proteins adopted a dimeric conformation.

### Phosphomimetic mutation T105D reduced the protein stability of AtGTR1 and disrupted its GLS transport

**Figure 7:**
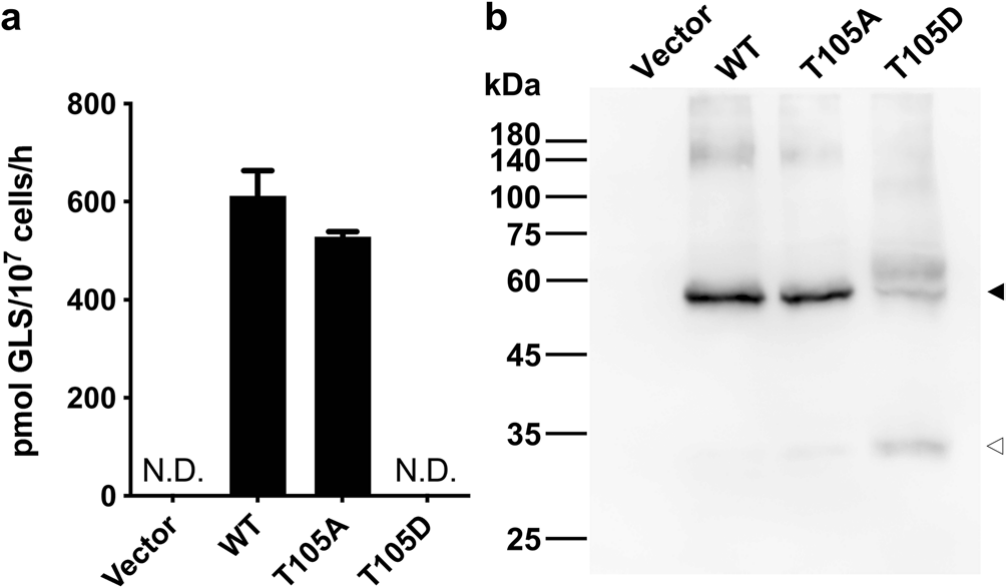
Phosphomimetic mutant of AtGTR1 lost GLS transport function and protein expression in yeast. (**a**) GLS transport activity of T105 mutants was determined by yeast uptake assay. The GLS uptake was quantified by RP-HPLC and showed as vertical bars with errors (mean ± SD, n = 3); N.D. indicates nondetectable. (**b**) Western blot of yeast cells expressing AtGTR1 and its T105 mutants. An anti-His-tag antibody was used as the primary antibody. The filled triangle indicates the AtGTR1 monomer, whereas the open triangle indicates degraded AtGTR1.

It was known that phosphorylation of Thr101 of NPF6.3 suppressed the protein dimerization and converted the binding affinity of nitrate to high affinity^19,21^. This threonine residue is conserved in many NPF members, including AtGTR1 (Fig. S1)^21^. To determine whether AtGTR1 is also regulated by phosphorylation at this threonine residue, T105A and T105D mutants were established to mimic constitutively dephosphorylated and constitutively phosphorylated AtGTR1, respectively. These mutants were then used to assay their GLS transport activity. Comparison of the GLS transport activities showed that transport function was completely lost in the T105D mutant, while the T105A mutant showed transport activity similar to that of WT AtGTR1 (Fig. 7a). To further examine whether the decreased activity of the two mutants was related to protein expression levels, the yeast membranes of both mutants were isolated and analyzed by western blot with anti-His-tag antibodies (Fig. 7b). We found that the T105D mutant was expressed at lower levels than the WT and T105A mutant proteins, indicating the decrease in transport activity might be due to the reduced protein expression/stability (Fig. 7b). In addition, a degradation band close to 30 kDa was observed in the T105D membrane but not in the WT and T105A mutants, suggesting that protein stability may be influenced by T105 phosphorylation. These results indicate that Thr105 phosphorylation of AtGTR1 drastically affects its protein properties on the yeast membrane, leading to the loss of GLS transport function.

## Discussion

Through expressing AtGTR1 in *S. cerevisiae,* we measured the GLS transport activity of AtGTR1 using a yeast uptake assay coupled with quantification by RP-HPLC in this study. Interestingly, AtGTR1 transported all three classes of GLSs, and the GLS transport rates were negatively correlated to the hydrophobicity of substrates. Using a competition assay, we found that the addition of JA, SA, and IAA could inhibit the GLS transport activity of AtGTR1, indicating that these essential phytohormones may be substrates or analogs of AtGTR1. This information on substrate preference provided clues to understand the substrate selective mechanism of AtGTR1.

For characterizing AtGTR1, the yeast GLS uptake assay was utilized to investigate the GLS transport activity under different pH environments. The pH profiling of AtGTR1 shows a strong pH dependency with a pH optimal around pH 5.5–6.5 (Fig. 1f). It is different from other POT family members, which showed an optimal pH range around pH 6.5–7.5^32,33^. The higher transport activity of AtGTR1 at a more acidic environment may be important to accelerate the GLS uptake from apoplast in *A. thaliana*^12^. Furthermore, treatment of uncouplers confirmed that proton gradient was used to drive the AtGTR1 function (Fig. S3). These results show the feasibility of this yeast uptake assay for studying GLS transport of AtGTR1. We then utilized this assay to investigate the substrate preference of AtGTR1 and found that the hydrophobicity of GLSs determined the transport rate of AtGTR1 (Fig. 2a). Kinetic analysis showed that AtGTR1 has higher V_max_ values for transporting GLSs with lower hydrophobicity, such as GRA. Interestingly, the K_m_ value for transporting GRA is higher than transporting GTR and GBS. Therefore, we concluded that AtGTR1 showed a higher substrate affinity to bind the GLSs with higher hydrophobicity, and the tight binding of GLS leads to a lower translocation rate. These results suggested that hydrophobicity of the aglycone side chains might decide the substrate preference of AtGTR1. Thus, the hydrophobic interactions between GLSs and AtGTR1 may play an important role in the broad substrate specificity. This hypothesis is supported by the fact that the crystal structure of NPF6.3 shows a hydrophobic cavity for binding nitrate ions^19^. The AlphaFold predicted model of AtGTR1 also showed a similar hydrophobic pocket in the bottom of central cavity (Fig. S4). Furthermore, the molecular docking simulation revealed that this hydrophobic pocket provides enough space to accommodate various types of GLSs and allow hydrophobic contacts with the aglycone side chains of GLSs. In addition, Jørgensen et al. have proposed that three hydrophobic residues in the central cavity, I52, I53, and L56, may contribute to determining the substrate specificity of GLSs^2^. Together, the interaction between the aglycone side chains of GLSs and the hydrophobic pockets of AtGTR1 may determine the transport rate of these GLSs.

Furthermore, we conducted the phytohormone competition experiments and found that treatment with JA, SA, and IAA inhibited AtGTR1 from transporting GLS, whereas treatment with ABA or GA did not. It is expected that JA could compete with GLS due to that JA and JA-Ile have been reported as the substrates of AtGTR1^11^. Surprisingly, SA treatment also inhibited GLS transport in yeast. Although SA is not reported to be transported via any NPF transporter, including AtGTR1, the inhibitory curve showed the IC_50_ of SA even lower than JA, suggesting SA may also bind to AtGTR1. SA and JA are both phytohormones involved in the regulation of plant immune responses^34^. Interestingly, the application of JA and SA to *Arabidopsis* and *Brassica* has been shown to increase the accumulation of GLSs^35–37^. Furthermore, recent studies showed that SA treatment stimulated the gene expression of GTR1 in Chinese kale^38^. These studies suggest that SA may be able to serve as a substrate or analogues of AtGTR1. Moreover, IAA revealed a cooperative inhibition on GLS transport of AtGTR1 (Fig. 4c), and the competition kinetic analysis further confirmed the non-competitive properties of IAA (Fig. S2). IAA has been reported as a substrate for NPF6.3 transporter^39^. Previous studies also mentioned that IAA signaling regulates biosynthesis of GLS and affects GLS level in *Arabidopsis*^40,41^. Besides, GLS metabolically involved in auxin homeostasis, and interruption of GLS metabolism would result in a high-auxin phenotypes^41^. Taken together, these data suggest that IAA may be involved in the regulation of GLS transport by modulating the activity of AtGTR1. On the other hand, despite being a known substrate of AtGTR1, GA does not affect GLS transport. This may be since AtGTR1 transports GA at a slower rate than it transports GLS^10,22^. Besides, it is also possible that AtGTR1 transports GA through another pathway, different from the GLS. These competition experiments revealed that JA, IAA, and SA might serve as substrates and/or analogs of AtGTR1. More evidence from biochemical and physiological studies is needed to clarify the functional roles of these phytohormones on AtGTR1 transport activity.

In addition to exploring the substrate preference of AtGTR1, we focused on studying the functional residues in the central cavity of AtGTR1. The mutagenesis studies confirmed that four charged residues, Glu45, Glu48, Lys49, and Arg166, are essential for GLS transport (Fig. 4a). Interestingly, in our study, the E48A mutant retained a very small amount of GLS transport activity, whereas E45A and K49A lost GLS transport function completely (Fig. 4a). The residual activity of E48A suggested that Glu48 may play a different role from Glu45 in the proton coupling mechanism. Outside of this motif, another mutant, R166A, completely lost its GLS transport function. This positively charged residue on TMH 4 has been proposed to interact with a dipeptide substrate in POT protein^3,42^. In this work, the loss of function of the R166A mutant supports that Arg166 may participate in capturing GLSs during the transport cycle. Molecular docking simulations also indicate that Arg166 may bind to the sulfate group of GLSs. These mutagenesis experiments suggested that the proton coupling and substrate binding mechanisms of AtGTR1 are similar to other POT proteins.

Moreover, it is known that protein conformation is essential for regulating the function of membrane transporters^43^. For example, the lactose transporter, LacS, forms a dimer with cooperative activities^44^; and the dual-affinity nitrate transporter, NPF6.3, showed high nitrate affinity as a monomer while forming a homodimer with low affinity^21^. Here, we revealed that the AtGTR1 purified from *S. cerevisiae* adopts a homodimeric conformation. However, the functional significance of the AtGTR1 dimerization remains unclear because the decoupled AtGTR1 has not been functionally characterized. Unlike that phosphorylation of NPF6.3 induced protein dissociation^21^, phosphorylation of AtGTR1 caused protein degradation at yeast membrane (Fig. 7b). A recent study has also reported that phosphorylation in AtGTR1 regulates the plasma membrane localization in *Xenopus oocytes*^22^. Thus, we speculate that phosphorylation of AtGTR1 may lead to dimer dissociation and further result in reduced stability. Nevertheless, it remains to be confirmed whether this phosphorylation of AtGTR1 occurs in plants. Proteomic analysis of the post-translational modifications on AtGTR1 in *Arabidopsis* would be helpful to understand the regulation of AtGTR1 and GLS signaling.

This study showed that JA, IAA, and SA could compete with GLS for the transport function of AtGTR1 in *S. cerevisiae*. It is important to further investigate how these phytohormones affect the GLS transport in plants. Previous studies have shown that AtGTR1 is involved in JA signaling and stress response^10,11,45^. Monitoring the changes on the SA and IAA signaling in the *gtr1 gtr2* knockout plants may provide hints to understand the crosstalk between these phytohormones. Also, mutant plants defective in salicylic acid induction could be used to study GLS distribution and monitor the AtGTR1 expression to further reveal the function of SA on GLS signaling^35^. In addition, the homodimeric conformation of AtGTR1 has not yet been reported in plant cells, and it is also no clear whether AtGTR1 could form heterodimers with other NPF proteins. Transient expression of AtGTR1 in leaves coupled with split-GFP association assay may be able to elucidate the interactions of AtGTR1 dimer^46^. In addition, protein structural studies will be helpful to elucidate the mechanism of broad selectivity of AtGTR1. Solving the structure of AtGTR1 binding to phytohormone molecules will provide solid evidence to elucidate the molecular mechanism. Currently, we have established the purification protocols to isolate the AtGTR1 proteins from the yeast membrane (Fig. 6b). These protocols could be further utilized to prepare purified AtGTR1 for protein crystallography and cryo-EM studies.

Additionally, the phytohormones that can inhibit AtGTR1 transporting GLS have the potential to act as growth regulators to control GLS levels in crops. A recent study showed that knockout of GTR1 orthologs reduced seed GLS contents in *Brassica rapa* and *Brassica juncea*^47^. Therefore, we hypothesize that the treatment of inhibitors and analogs of GTR1 may also reduce the GLS contents in plants. These results increased our knowledge of the plant transporters regulating the distribution of defense metabolites and contributed to the development of modern agriculture.

## Materials and methods

### Chemicals

All glucosinolates and phytohormones used in this study is purchased from suppliers. The glucosinolates include sinigrin (allyl glucosinolate, PN#S0156, Tokyo Chemical Industry, Japan), gluconapin (3-butenyl glucosinolate potassium salt, PN#89688, phytolab, U.S.A.), glucoerucin (4-(methylthio)butyl glucosinolate potassium salt, PN#89686, phytolab, U.S.A.), glucoraphanin (4-(methylsulfinyl)butyl glucosinolate potassium salt, PN#89,215, phytolab, U.S.A.), sinalbin (p-hydroxybenzyl glucosinolate potassium salt, PN#89793, phytolab, U.S.A.), glucotropaeolin (benzyl glucosinolate potassium salt, PN#G0397, Tokyo Chemical Industry, Japan), and glucobrassicin (3-indolylmethyl glucosinolate potassium salt, PN#80593, phytolab, U.S.A.). The phytohormones includes IAA (3-indoleacetic acid, PN# I2886, Sigma-Aldrich, U.S.A.), SA (salicylic acid, PN#1901-2150, Showa Chemical Industry, Japan), JA (jasmonic acid, PN#J0004, Tokyo Chemical Industry, Japan), GA (gibberellin A3, PN#G0029, Tokyo Chemical Industry, Japan), ABA ((±)-abscisic acid, PN#A1049, Sigma-Aldrich, U.S.A.).

### Plasmid construction and mutagenesis

The plasmid construction methods were based on previous studies with some modifications^48^. The cDNA encoding AtGTR1 with a C-terminal 8-His-tag was synthesized and inserted into the pESC-URA vector (Agilent Technologies, U.S.A.) between the restriction sites, *Bam*HI and *Xho*I. To express AtGTR1-GFP, AtGTR1 cDNA was subcloned into the pDDGFP-2 vector (a kind gift from Dr. David Drew at Stockholm University) between the restriction sites, *Bam*HI and *Xma*I. All the mutants used in this study were generated by using the In-Fusion^®^ HD Cloning Kit (Takara Bio, U.S.A.) and confirmed by DNA sequencing^49^. The PCR primer pairs used to generate mutants are listed in Supplementary Table S2.

### Yeast transformation and expression

The plasmid constructs were transformed into the *S. cerevisiae* strain FGY217 (MATa, ura3-52, lys2Δ201, and pep4Δ; a kind gift from Dr. David Drew at Stockholm University) by the LiAc/polyethylene glycol method^50^. The yeast transformants were cultured in complete minimal (CM) medium (1.7% (w/v) yeast nitrogen base, 5% (w/v) ammonium sulfate, 2% (w/v) glucose, and 0.01% (w/v) l-lysine; pH 5.5) at 30 °C for 18 h. Some of the overnight cultures were stored at − 80 °C with the addition of 15% glycerol, while the rest were diluted in fresh CM medium and cultured at 30 °C until the OD_600_ reached 1.0. To induce protein expression of AtGTR1, the medium was replaced with CM-galactose containing 2% (w/v) galactose instead of glucose, and the yeast cells were induced at 30 °C for 48 h.

### Yeast cell uptake assay and phytohormone competition assay

Yeast cells expressing AtGTR1 were washed twice and resuspended in a fresh CM medium containing 20 mM sodium citrate at pH 5.5. The cell density was determined and adjusted to 1 × 10^8^ cells/ml based on OD_600_ values. One milliliter of the cells was then harvested and resuspended in 1 ml of CM medium containing 20 mM sodium citrate and 100 µM GLSs with three replicates per condition. The uptake of GLSs by yeast cells was carried out at 30 °C for 1 h and terminated by centrifugation of the yeast cells at 6000×*g* for 5 min. The harvested yeast cells were immediately lysed for subsequent GLS quantification as described in the next section. For K_m_/V_max_ determination, GRA, GTR, and GBS were selected as the substrate and diluted to 8 concentrations (2.5, 5, 10, 20, 40, 80, 160, and 320 μM) in the assay medium. Three replicates were prepared for each condition. The uptake rate was measured at an incubation time of 15 min.

In the phytohormone competition experiments, one milliliter of 1 × 10^8^ cells/ml yeast cells expressing AtGTR1 were harvested and resuspended in 1 ml CM medium containing 20 mM sodium citrate, 25 μM GTR, and 500 μM of each phytohormone. Three replicates were prepared for each experimental condition. These samples were then incubated at 30 °C for 1 h and terminated as mentioned above. After wash by fresh CM medium twice, the yeast cells were harvested by centrifugation and lysed immediately for GLS quantification as desulfo-GLS by HPLC as described in the following section. In addition, to obtaining the competition curves of JA, IAA, and SA, the following concentrations of phytohormones were used to compete with 25 μM GTR: 0, 62.5, 125, 250, 500, 1000, 2000 μM. Three replicates of each concentration were tested. Other experimental steps were the same as the above competition experiments to acquire the transport rate of GTR under different phytohormone concentrations.

### GLS quantification as desulfo-GLS by RP-HPLC–DAD

The GLS quantification procedures were performed according to previous studies with modifications^51,52^. The yeast cells that had taken up the GLSs were resuspended in 70% methanol containing 1 µM GNA as the internal standard and then incubated at 75 °C for 10 min for cell lysis. After centrifugation at 4000×*g* for 10 min to remove the insoluble precipitates, the supernatant was loaded onto 0.25 ml of DEAE Sephadex™ A-25 resin (Cytiva, U.S.A.), which was activated and pre-equilibrated with 70% methanol. After sample binding, the resin was washed twice with 70% methanol and distilled water. The resin was then washed with desulfation buffer (20 mM MES, pH 5.2). The residual buffer was removed from the resin by centrifugation at 1000×*g* for 1 min, and 125 µl of desulfation buffer containing 0.125 U sulfatase (PN#S9626, Sigma-Aldrich, U.S.A.) was added to fully rinse the resin. The resin was then incubated at 25 °C for 18 h in the dark to release the GLSs from the resin by removing their sulfate group. The buffer containing desulfo-GLSs were eluted from the resin by centrifugation at 1000×*g* for 1 min and quantified by using an Agilent 1260 HPLC system with a C18 column (Poroshell 120 EC-C18, 3.0 × 100 mm, 2.7 µm, Agilent Technologies, U.S.A.). The injection volume is 80 µl. The mobile phase consisted of solvent A (distilled water) and solvent B (methanol), with a linear gradient of 1–19% solvent B over 25 min. The flow rate was 0.8 ml/min, and the column temperature was 30 °C. The UV absorbance was monitored at 229 nm. The peak area of each GLS was integrated by the Openlab ChemStation program and corrected by using the internal standard peaks. The standard curve of each type of GLS was established by using 0.25, 0.5, 1, 2, 4, 8, and 16 µM samples. Both standards and samples were subjected to similar procedures of solid phase extraction and desulfation as mentioned earlier.

### Yeast microsomal membrane isolation

The method used to isolate yeast microsomal membranes was carried out as described in previous studies with minor modifications^53^. Yeast cells that expressed AtGTR1 or AtGTR1-GFP were washed with yeast wash buffer (0.1 M Tris–HCl (pH 9.4), 7 mM 2-mercaptoethanol) and then incubated in Zymolyase medium (100 mM Tris–HCl, 1% (w/v) yeast extract, 2% (w/v) peptone, 1% (w/v) glucose, 700 mM sorbitol, 5 mM 2-mercaptoethanol, 30 mg/ml Zymolyase; pH 8.0) at 30 °C for 3 h. The yeast spheroplasts were collected by centrifugation, resuspended in lysis buffer (50 mM Tris-ascorbate, 5 mM EGTA-Tris, 10% (w/v) glycerol, 1.5% (w/v) polyvinylpyrrolidone 40,000 (PVP40000), 1 mM pepstatin A, and 1 mM phenylmethanesulfonyl fluoride (PMSF); pH 7.6) and homogenized by ultrasonication on ice. The sonication intensity was limited to 960 J/mL using an ultrasonic generator (UP200S, Hielscher, Germany). After removing the cell debrides by centrifugation at 4000×*g* for 10 min, the supernatant was ultracentrifuged at 100,000×*g* for 60 min at 4 °C. The microsomal membranes in the pellet were resuspended in storage buffer (50 mM Tris–HCl, 400 mM NaCl, 20% (w/v) glycerol; pH 7.6) and ultracentrifuged again to remove the residual soluble and peripheral membrane proteins. The microsomal membranes were resuspended in storage buffer for subsequent protein purification.

### AtGTR1 purification and SEC analysis

The membrane protein solubilization and purification methods were as described in previous studies with optimization for AtGTR1^53^. The yeast microsomal membrane containing AtGTR1 or AtGTR1-GFP was diluted with storage buffer (50 mM Tris–HCl, 400 mM NaCl, 20% (w/v) glycerol; pH 7.6) containing 1% (w/v) n-Dodecyl β-d-maltoside (DDM) to a final protein concentration of 3 mg/ml and gently stirred at 4 °C for 60 min. After solubilization, the samples were ultracentrifuged at 100,000×*g* for 1 h at 4 °C. The supernatant was purified on a Ni–NTA column with an FPLC system (ÄKTA pure, Cytiva, U.S.A.). After washing the column with 50 mM imidazole, AtGTR1 was eluted with IMA buffer (50 mM Tris–HCl, 400 mM NaCl, 20% (w/v) glycerol, 0.03% (w/v) DDM, 0.03% (w/v) sodium cholate, 1 mM pepstatin A; pH 7.6) containing 300 mM imidazole. The purified AtGTR1 was dialyzed against SEC buffer (20 mM Tris–HCl, 150 mM NaCl, 10% (w/v) glycerol, 0.03% (w/v) DDM, 1 mM pepstatin A; pH 7.6) and concentrated for SEC. SEC is also conducted by using an FPLC system, and 1 mg of purified AtGTR1 was injected into a Superdex S200 pg 10/600 column and eluted with SEC buffer at 4 °C. The fractions containing dimeric AtGTR1 were collected and concentrated for further analysis.

### Statistical analysis

Significant differences between two groups were assessed by a two-tailed unpaired Student t-test. Multiple group comparisons were performed by one-way ANOVA followed by Tukey’s multiple comparisons test. The K_m_ and V_max_ values and associated standard errors were obtained by nonlinear regression estimation to fit the Michaelis–Menten equation. The IC_50_ value was calculated by using nonlinear regression estimation to fit the [inhibitor] versus normalized response equation with variable Hill slope; no curve fitting restraints were included.

## Supplementary Information

Supplementary Information.
