# Supplementary material for: Transport efficiency of AtGTR1 dependents on the hydrophobicity of transported glucosinolates
Source: Sci Rep. 2022 Mar 24;12:5097. doi: 10.1038/s41598-022-09115-x (PMC8948214; doi:10.1038/s41598-022-09115-x)
Supplement: Supplementary file 1 — Supplementary Information. [file 41598_2022_9115_MOESM1_ESM.pdf]

Transport Efficiency of AtGTR1 Dependents on the Hydrophobicity of Transported Glucosinolates

Yi-Chia Chung[1], Hao-Yu Cheng[1], Wei-Tung Wang[1], Yen-Jui Chang[1], Shih-Ming Lin[1,2*]

[1] *Department of Biotechnology and Bioindustry Sciences, National Cheng Kung University*

[2] *Institute of Tropical Plant Sciences and Microbiology, National Cheng Kung University*

**Correspondence: smlin@mail.ncku.edu.tw*

Supplementary Information

Supplementary Table S1. V_{max} and K_m values of AtGTR1 transporting GLS against a range of IAA concentrations

Competitors	Correlation coefficient (R^2)	V_{max} (pmol/10^7 cells/h)*	K_m (μM)*
0 μM IAA	0.9478	3224.4 ± 107.96	28.44 ± 3.373
250 μm IAA	0.9696	2795.6 ± 67.25	25.48 ± 2.245
500 μm IAA	0.9452	2247.5 ± 72.00	25.46 ± 2.988
1000 μm IAA	0.9693	1395.1 ± 41.28	36.58 ± 3.557
2000 μm IAA	0.9687	658.5 ± 19.92	37.13 ± 3.675

Correlation coefficient indicates the extent to which the kinetic curves were fitted by the nonlinear regression. *V_{max} and K_m values are mean ± SE, n = 18 (6 substrate concentrations * 3 independent repeats)

Supplementary Table S2. Primers used for construction of AtGTR1 mutants

Mutants		Sequence (5' to 3')
E45A	Forward	GGTAATG**CA**ACATTTGAGAAGCTTGGG
	Reverse	AAATGT**TG**CATTACCAATGATAAAAGGCATG
E48A	Forward	ACATTTG**CA**AAGCTTGGGATCATAGGG
	Reverse	AAGCTT**TG**CAAATGTCTCATTACCAATGATA
K49A	Forward	TTTGAG**GCT**CTTGGGATCATAGGGACATTATCA
	Reverse	CCCAAG**AGC**CTCAAATGTCTCATTACCAATG
T105A	Forward	TACAAG**G**CTCTCAGTGTCGCTGTCATCGC
	Reverse	ACTGAGAG**C**CTTGTAGCGACCAAAGTAAGTGTCG
T105D	Forward	TACAAG**GA**TCTCAGTGTCGCTGTCATCGCT
	Reverse	ACTGAGA**TC**CTTGTAGCGACCAAAGTAAGTGTCGC
R166A	Forward	GGTATC**GC**ACCGTGTAATTTAGCGTTTGG
	Reverse	ACACGGT**GC**GATACCACCAGCGCC

The mutated codon are underlined and the replaced nucleotides are shown in bold.

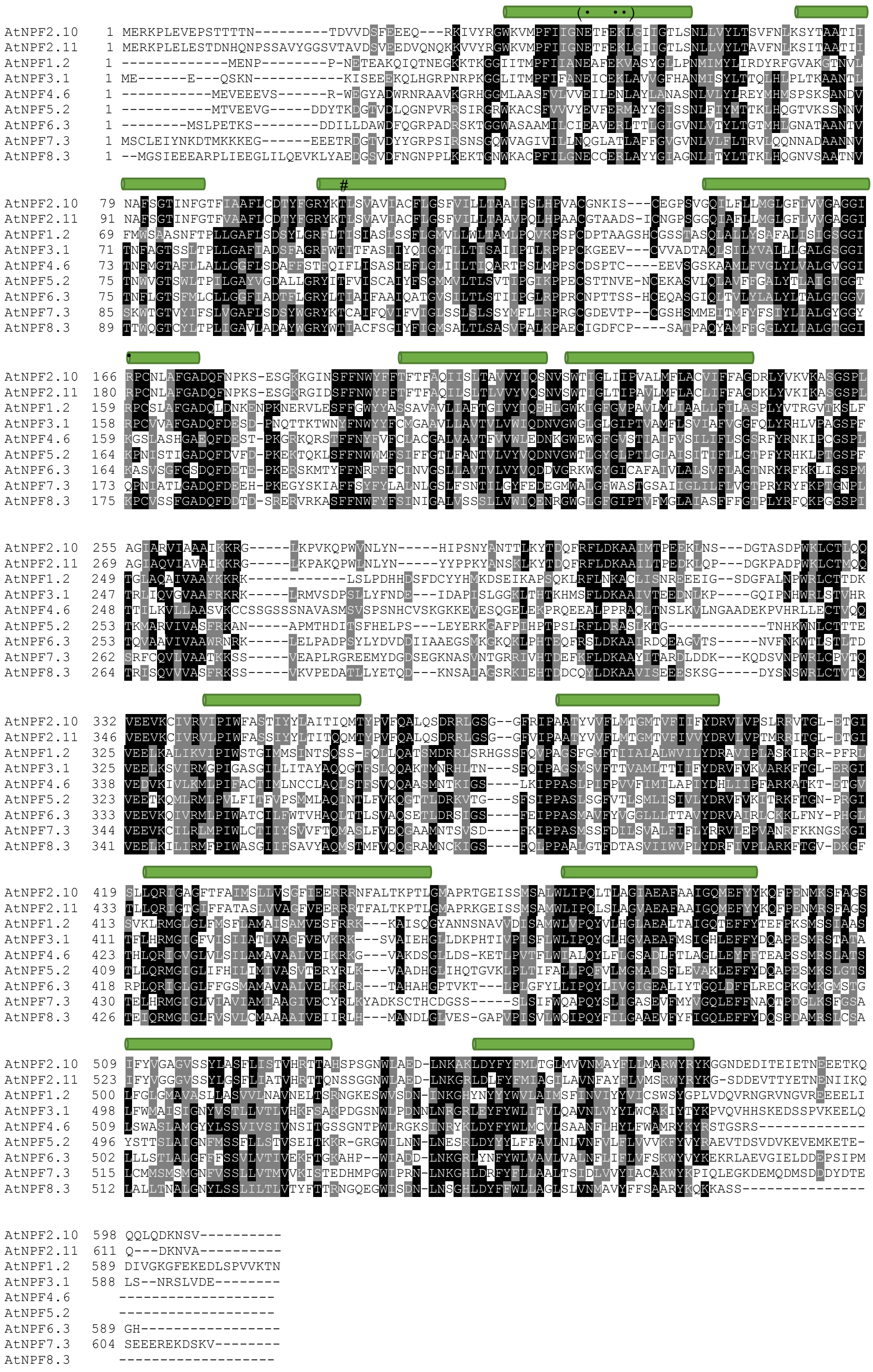

Supplementary Figure S1. Sequence alignment of plant NPF family. Strictly conserved residues are colored in black box. Gray box indicates the consensus residues. Green cylinders showed the transmembrane helixes. • means the charged residues investigated in this study. # labeled the phosphorylation site known to regulate the dimerization of NPF6.3. The ExxEK motif were specified by a bracket.

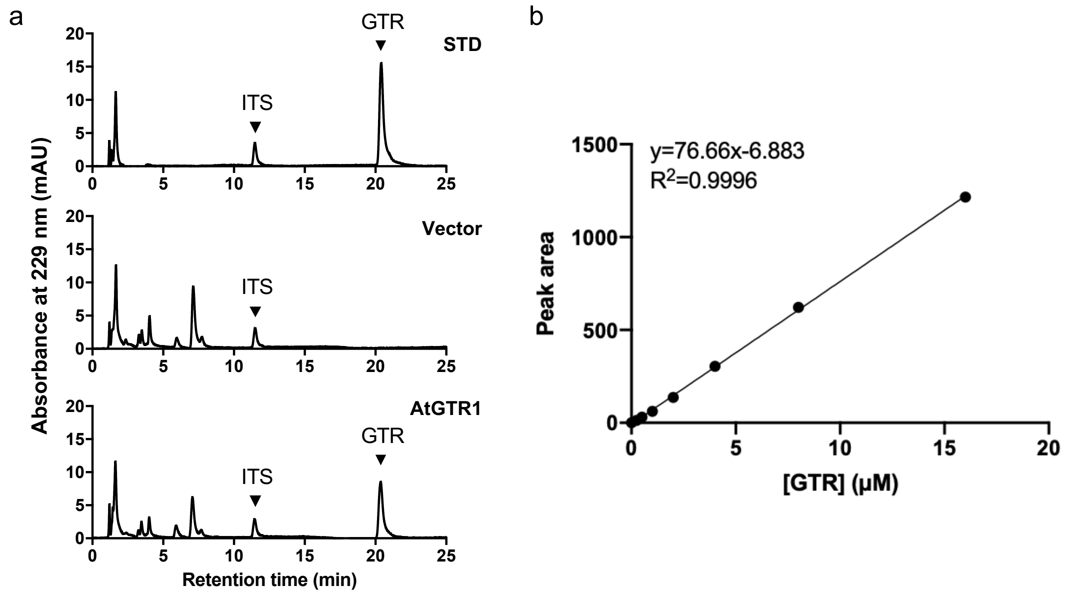

Supplementary Figure S2. GLS quantification by RP-HPLC showed a clear background and good linearity. (a). Chromatograms of Upper panel: 4 μM glucotropaeolin (GTR) as the standard (STD); Middle and Bottom panel: yeast cells expressing vector and AtGTR1 were incubated with 100 μM GTR for 1h, respectively. All samples were spiked with 1 μM gluconapin (GNA) as the internal control (ITS) and then treated with solid-phase extraction and desulfation for RP-HPLC quantification. (b) Calibration curve were plotted by peak areas against concentration of GTR standards. The GTR standards were prepared by a two-fold serial dilution in the range of 0.25-16 μM. The linear regression equation and correlation coefficients (R^2) were calculated and shown in the figure.

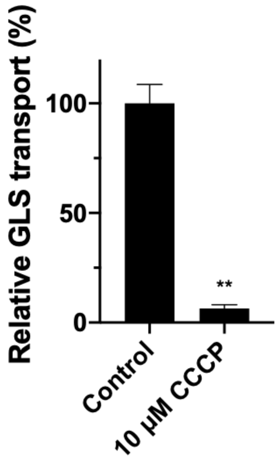

Supplementary Figure S3. The GLS transport activity of AtGTR1 depends on the proton gradient. The uncoupler, CCCP, was added in the uptake medium for measuring the GLS transport activity by yeast uptake assay. The uptake rate is normalized to control group. ** represents $p<0.01$. Bar values and error bars indicate mean ± SD. (n=3)

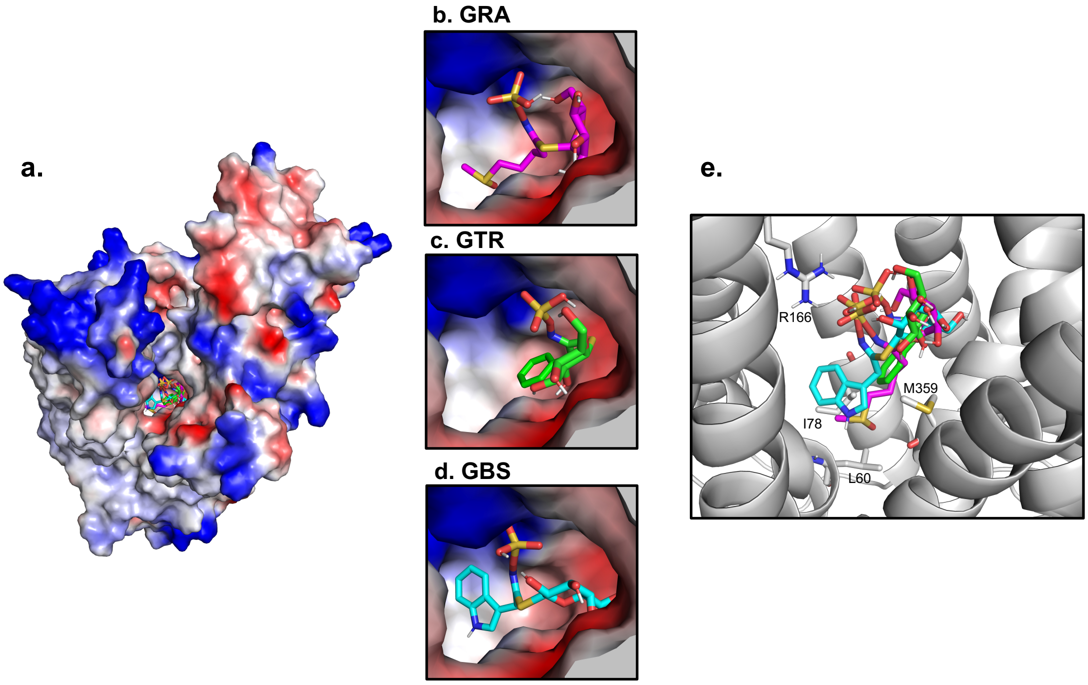

Supplementary Figure S4. AutoDock-mediated docking models of AtGTR1 showed the aglycone side chains of GLSs interact with the hydrophobic regions in the central cavity. The AlphaFold-predicted model of AtGTR1 (ID: AF-Q944G5-F1) was docked with the molecular models of GRA (magenta), GTR (green) and GBS (cyan), respectively. (a). The molecular surface of predicted AtGTR1 showed an inward-open conformation. All GLSs molecules which represented as sticks are docked in central cavity. The surface gradient colors indicates the charge distribution (red: negative-charged; blue: positive-charged; white: neutral regions). (b-c) The zoom-in views of the central cavity docked with (b) GRA, (c) GTR and (d) GBS. The binding energies for GRA, GTR and GBS are -4.05, -5.07 and -6.18 kcal/mole, respectively. (e) The docked GLSs interact with several residues in the central cavity. The AtGTR1 is displayed as cartoon ribbon. The positive-charged Arg166 and three hydrophobic residues interacting with the docked GLSs are labeled and shown in sticks.

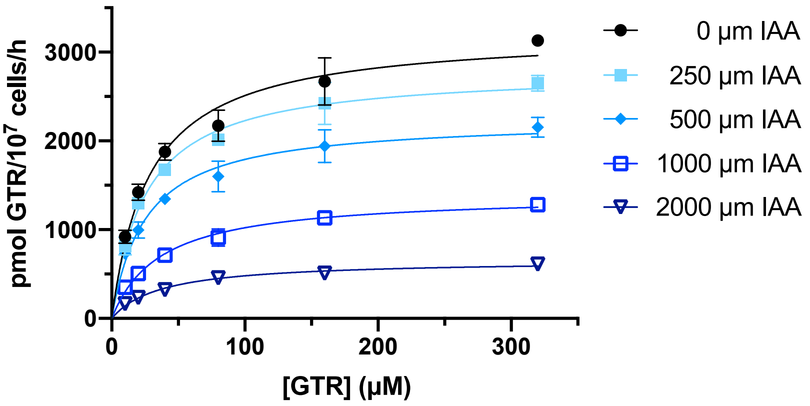

Supplementary Figure S5. Competition kinetics of AtGTR1 transporting GLSs against various concentrations of IAA showed a mixed mode competition properties. IAA were spiked in the uptake medium to a final concentration of 250, 500, 250 and 1000 for measuring the kinetic curves of AtGTR1 transporting GTR. All kinetic curves was measured by yeast uptake assay at substrate concentrations in the range of 10-320 µM with a 2-fold serial dilution. Each data point represent mean ± SD (n =3). The correlation coefficient, K_m and V_{max} values were shown in Supplementary Table S1.

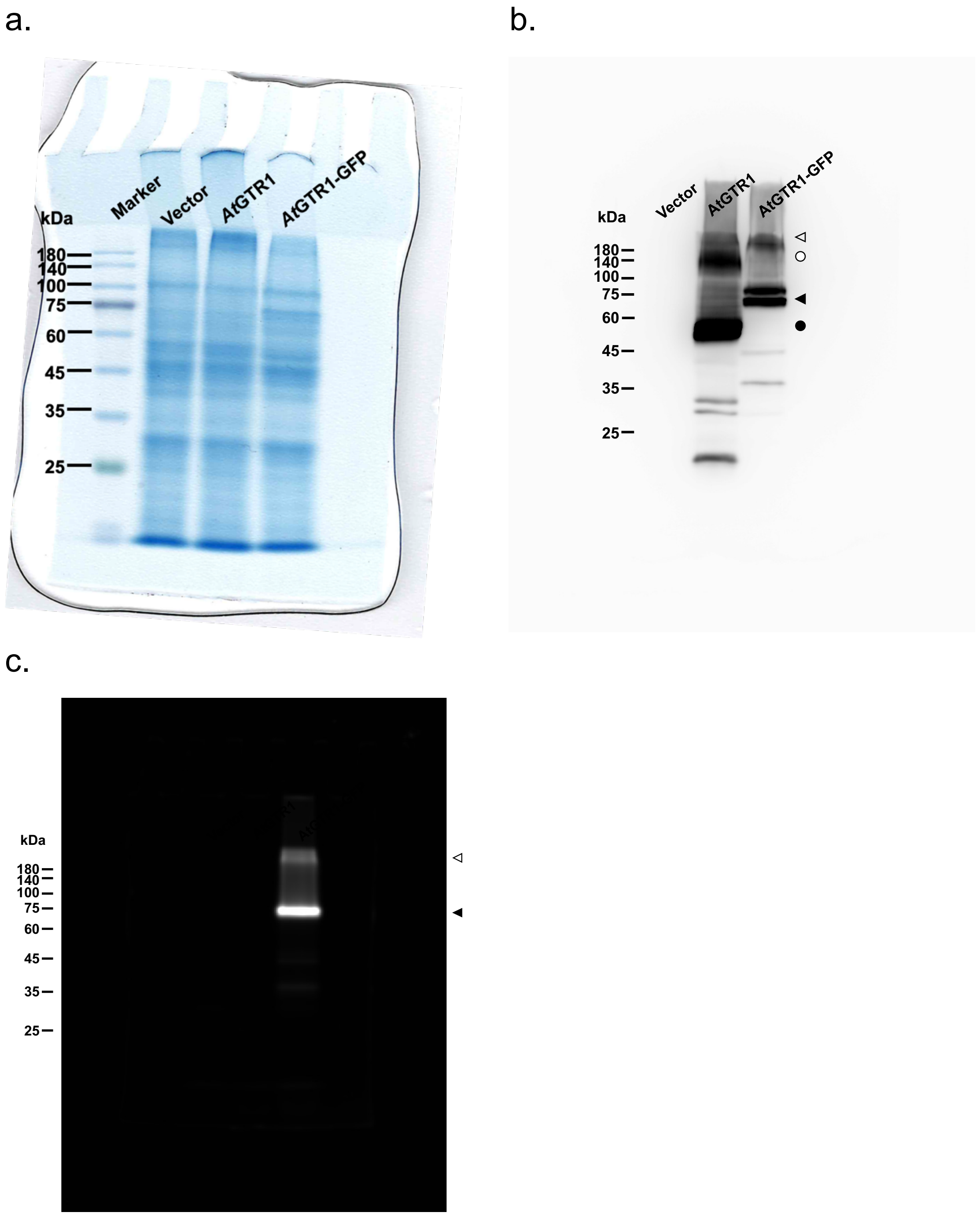

Supplementary Figure S6. Uncropped, full length (a) SDS-PAGE, (b) western blotting, and (c) in-gel fluorescence for Figure 1.

a.

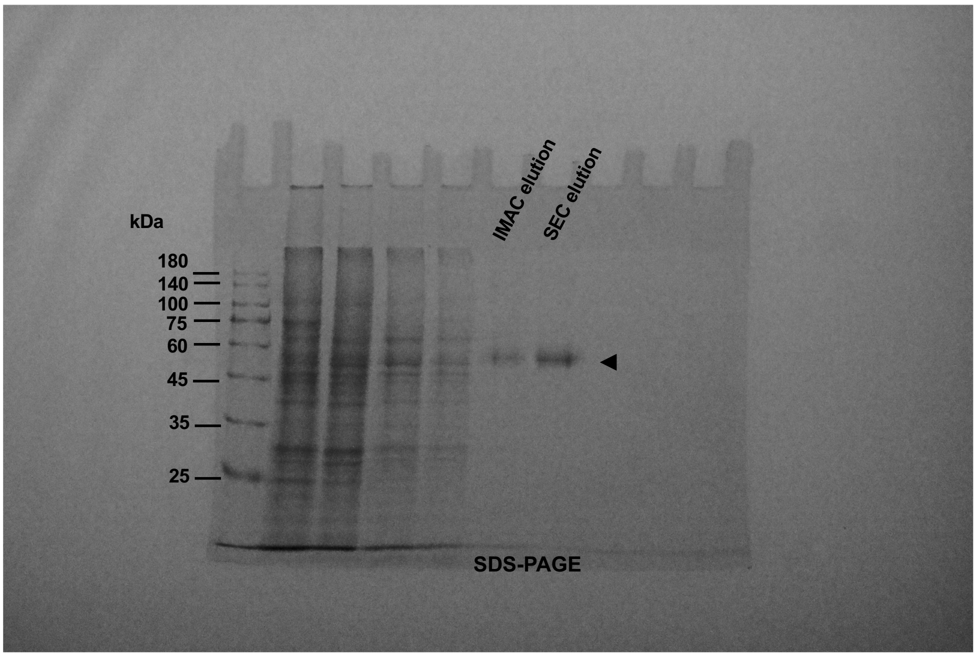

b.

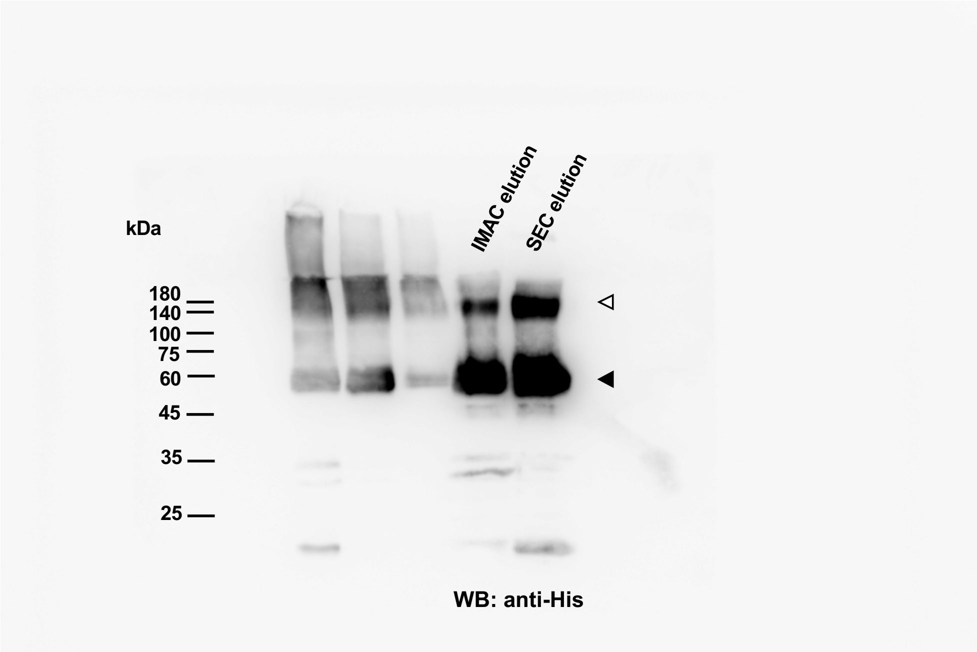

Supplementary Figure S7. Uncropped, full length (a) SDS-PAGE and (b) western blotting for Figure 5.

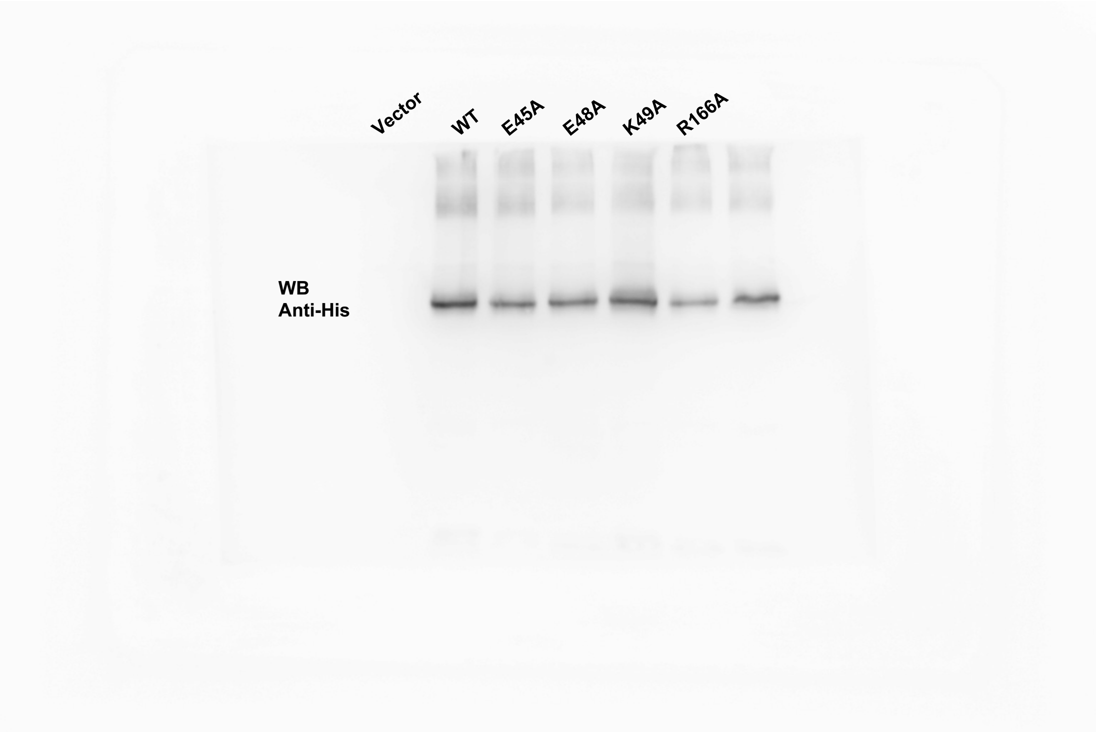

Supplementary Figure S8. Uncropped, full length western blotting for Figure 6.

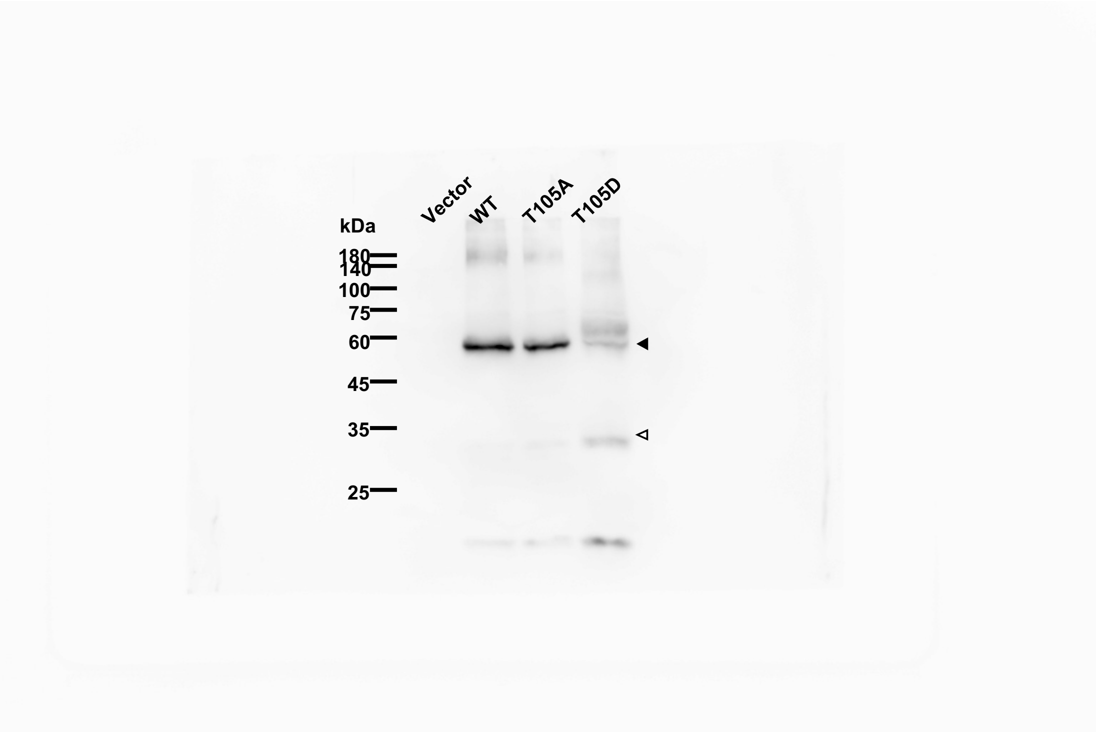

Supplementary Figure S9. Uncropped, full length western blotting for Figure 7.

Supplementary Materials & Methods

Yeast uptake assay with CCCP uncoupler treatment

The treatment methods of carbonyl cyanide m-chlorophenyl hydrazone (CCCP) for uncoupling proton gradient is according to previous study with minor modification[1]. Yeast cells expressing AtGTR1 were harvested after induction and washed twice with uptake medium (1.7% (w/v) yeast nitrogen base, 5% (w/v) ammonium sulfate, 20 mM sodium citrate, pH 5.5). The 1 x 10^8 yeast cells were collected and resuspended with 1 ml fresh uptake medium containing 160 μM GTR and 10 μM CCCP (C2759, Sigma-Aldrich, U.S.A.). After fully resuspended, the yeast cells were incubated for at 30°C for 1 h to accumulate the GTR, and then harvested by centrifugation at 6,000 x g for 5 min. The yeast cells were washed with 1 ml fresh uptake medium for twice and lysed by addition of 70% methanol containing 1 μM GNA as the internal standard and incubated at 75°C for 15 min. The GTR content in the yeast lysates were further determined by RP-HPLC as described in the Material & Methods.

IAA competition kinetics analysis

To study the IAA competition kinetics, yeast uptake assay were employed to determine the K_m and V_{max} of AtGTR1 under different concentration of IAA. The uptake medium (1.7% (w/v) yeast nitrogen base, 5% (w/v) ammonium sulfate, 20 mM sodium citrate, pH 5.5) containing 0, 250, 500, 1000 and 2000 μM IAA were prepared for dilution of substrates. The substrate GTR was serially diluted to final concentrations of 10, 20, 40, 80, 160 and 320 μM in the uptake medium with various IAA concentrations. The AtGTR1-expressing yeast cells were harvested and washed by fresh uptake medium by centrifugation at 6,000 x g for 5 min. The 1 x 10^8 yeast cells were resuspended in 1 ml uptake medium with various IAA and GTR concentration, and three replicates were prepared for each condition. The uptake rate of the yeast cells was measured at an incubation time of 15 min at 30°C. After incubation, the samples were centrifugated at 6,000 x g for 5 min and washed with 1 ml fresh uptake medium for twice. The cells were immediately resuspending in 1 ml 70% methanol containing 1 μM GNA. The samples were incubated at 75°C for 15 min for cell lysis and then used to determine the GTR content by RP-HPLC as described in the Material & Methods.

Molecular docking of glucosinolates into AtGTR1

Molecular docking was performed using AutoDock 4.2 and the docking methods are based on previous studies with minor modifications[2,3]. The predicted protein model of AtGTR1 was grabbed from AlphaFold database (ID: AF-Q944G5-F1)[4,5], and the unstructured N-terminal and C-terminal regions were removed for following docking

simulation. The molecular 3D models of GLSs were downloaded from KNApSAcK-3D database[6]. Non-polar hydrogens and Gaisteiger charges were added to both protein and ligand models by AutoDockTools[2]. The number of torsions for GRA, GTR and GBS are set to 15, 12 and 12, respectively. Search spaces were defined as a 60 × 60 × 60 points grid box with a point spacing of 0.375 Å and centered on the central cavity near the Lys49 and Arg166 of AtGTR1. A Lamarckian genetic algorithm was used to search for 50 docking poses for each GLS. The population size is set at 300. The docking results were exported by AutoDockTools[2] and displayed by PyMOL.

Supplementary references

1. Liu, T. Y. *et al.* Identification of plant vacuolar transporters mediating phosphate storage. *Nat. Commun.* **7**, 1–11 (2016).
2. Morris, G. M. *et al.* AutoDock4 and AutoDockTools4: Automated docking with selective receptor flexibility. *J. Comput. Chem.* **30**, 2785–2791 (2009).
3. Forli, S. *et al.* Computational protein-ligand docking and virtual drug screening with the AutoDock suite. *Nat. Protoc.* **11**, 905–919 (2016).
4. Jumper, J. *et al.* Highly accurate protein structure prediction with AlphaFold. *Nature* **596,** 583–589. (2021).
5. Varadi, M. *et al.* AlphaFold Protein Structure Database: massively expanding the structural coverage of protein-sequence space with high-accuracy models. *Nucleic Acids Res.* **50**, D439–D444 (2022).
6. Nakamura, K. *et al.* KNApSAcK-3D: A Three-Dimensional Structure Database of Plant Metabolites. *Plant Cell Physiol.* **54**, e4–e4 (2013).
